# GPR4 regulates temporally dynamic and sex-dependent cocaine-induced locomotor sensitization

**DOI:** 10.3389/fnmol.2026.1894955

**Published:** 2026-08-12

**Authors:** Eric S. Chai, Dylan Glaser, Joseph Cascone, Grant Brady, Abbas Bader, Christina Cacoulidis, Anshika Kapoor, Emily Yu, Yuyang Chu, Safal Sapkota, Sarah Ding, Xiang-Ping Chu

**Affiliations:** 1 Department of Biomedical Sciences, University of Missouri–Kansas City School of Medicine, Kansas City, MO, United States; 2 Department of Orthopedic Surgery, Wayne State University School of Medicine, Detroit, MI, United States

**Keywords:** cocaine, dopamine, GPR4, locomotor activity, neuroadaptation, sensitization, sex differences

## Abstract

**Background and objectives:**

Cocaine-induced locomotor activation and sensitization are widely used models for investigating dopaminergic signaling and neuroadaptive plasticity relevant to substance use disorders. G protein-coupled receptor 4 (GPR4) is a proton-sensing receptor implicated in pH-dependent cellular signaling, but its role in cocaine-induced behavioral responses remains unknown. Moreover, sex-dependent variability in stimulant responsiveness may interact with genotype in temporally dynamic ways that are poorly understood.

**Methods:**

Locomotor responses were examined in 166 three-month-old wild-type (WT) and GPR4 knockout (GPR4KO) mice. Mice received daily saline or cocaine (20 mg/kg, days 1–5) during repeated locomotor testing, followed by a 14-day abstinence period and a cocaine challenge (10 mg/kg) to assess expression of locomotor sensitization. Distance traveled was recorded in 5-min bins during 150-min sessions and analyzed using factorial ANOVA models with *post hoc* comparisons and effect size estimation.

**Results:**

Baseline locomotor activity during the initial treatment sessions was higher in GPR4KO mice than in WT mice and higher in females than in males. During acute cocaine exposure, GPR4KO mice exhibited enhanced locomotor responses relative to WT mice, with genotype effects being most pronounced during early treatment sessions and progressively attenuating by day 5. Sex differences showed a biphasic within-session pattern, with females displaying greater early locomotor activity and males exhibiting greater late-session activity. Following abstinence, overall pre-challenge baseline activity did not differ according to prior cocaine exposure; however, genotype effects on baseline activity were observed in a sex-and treatment-dependent manner. After the cocaine challenge injection, females exhibited greater locomotor responses than males. Sensitization effects were strongest during the early phase of the challenge session and became sex-dependent during later intervals, whereas genotype × sex × sensitization interactions emerged selectively during the late phase of the session.

**Conclusion:**

GPR4 modulates cocaine-induced locomotor activation and sensitization in a temporally dynamic and sex-dependent manner. These findings identify GPR4 as a previously unrecognized regulator of stimulant responsiveness and implicate proton-sensitive signaling mechanisms in the neurobehavioral adaptations associated with repeated cocaine exposure.

## 1 Introduction

Cocaine use disorder (CUD) remains a major public health challenge characterized by maladaptive neuroplasticity within mesocorticolimbic dopamine circuits (Thomas et al., 2008; Gu et al., 2010; Fernandez-Espejo and Rodriguez-Espinosa, 2011; Uys and Reissner, 2011; London et al., 2025). Behavioral sensitization, defined as the progressive enhancement of locomotor or motivational responses following repeated psychostimulant exposure, is widely used to investigate the neurobiological mechanisms underlying addiction-related plasticity (Hu and Becker, 2003; Steketee and Kalivas, 2011; Jung et al., 2013; Delage et al., 2023). Acute cocaine-induced locomotor activation reflects the immediate pharmacological effects of cocaine, whereas locomotor sensitization reflects enduring neuroadaptations that emerge during repeated exposure and abstinence (Breiter et al., 1997; Garavan et al., 2008; Santos-Baldaia et al., 2023).

Cocaine increases extracellular dopamine by blocking dopamine transporter-mediated reuptake, thereby enhancing dopaminergic neurotransmission within the nucleus accumbens and associated reward circuits (Kalivas and Stewart, 1991; Santos-Baldaia et al., 2023). Repeated cocaine exposure induces persistent neuroadaptations through coordinated dopaminergic, glutamatergic, and intracellular signaling mechanisms (Nestler, 2004). Dopamine D1 and D2 receptors, NMDA-and AMPA receptor-dependent synaptic plasticity, and cAMP/protein kinase A (PKA) and extracellular signal-regulated kinase signaling pathways regulate transcriptional programs mediated by factors such as CREB and ΔFosB (Carlezon et al., 1998; Savell and Hope, 2023), resulting in long-lasting structural and functional adaptations associated with addiction (Self and Nestler, 1995; Lu et al., 2006; Wolf and Ferrario, 2010; Lüscher and Malenka, 2011; Reissner, 2013; Samaha et al., 2021; Nestler, 2025). Consequently, identifying novel molecular regulators of acute cocaine responsiveness and behavioral sensitization may provide important insights into mechanisms underlying vulnerability or resilience to substance use disorders (Kelz et al., 1999; Robinson and Berridge, 2001; Nestler, 2004; Zachariou et al., 2006).

G protein-coupled receptor 4 (GPR4) is a proton-sensing G protein-coupled receptor (GPCR) activated by extracellular acidification that signals primarily through Gs/cAMP pathways, although coupling to additional signaling mechanisms has also been reported (Ludwig et al., 2003; Xue et al., 2006; Chen et al., 2011; Rowe et al., 2021; You et al., 2025; Wen et al., 2025). GPR4 has been implicated in the regulation of inflammation, vascular function, endothelial activation, and cellular stress responses (Dong et al., 2013; Dong et al., 2017; Krewson et al., 2020; Bai et al., 2025). While studies of GPR4 have focused predominantly on peripheral tissues, accumulating evidence indicates that GPR4 is also expressed within the central nervous system (Gonye et al., 2024), including the cerebrovascular endothelium and selected brain regions, where extracellular pH fluctuations can influence neuronal function under both physiological and pathological conditions (Bowers et al., 2004; Haque et al., 2020, 2021; He et al., 2024). More broadly, proton-sensing GPCRs regulate neuronal excitability, synaptic transmission, neuroinflammation, and neuromodulatory signaling, suggesting that proton sensing is an important contributor to neural circuit function (Bowers et al., 2004; Sanchez-Reyes et al., 2023; Bramlett et al., 2025; Singh et al., 2025; Li et al., 2026).

Cocaine-induced increases in dopaminergic signaling are accompanied by enhanced neuronal activity and metabolic demand within reward-related brain circuits (Porrino, 1993; Kalivas, 2007; Ciccarone and Shoptaw, 2022). These energetically demanding processes are associated with alterations in glucose utilization, lactate production, and transient shifts in extracellular pH that can modify the local ionic and metabolic microenvironment (Chesler, 2003; Bilodeau and Schwendt, 2016; Sanchez-Reyes et al., 2023; Clare et al., 2024). Consequently, proton-sensitive signaling mechanisms may represent an underrecognized component of cocaine-induced behavioral regulation. Because GPR4 is activated by extracellular acidification and primarily signals through cAMP pathways that are also implicated in cocaine-induced neuroplasticity, GPR4 may provide a mechanistic link between cocaine-induced neural circuit activation and pH-dependent neuromodulatory signaling. However, whether GPR4 contributes to acute cocaine responsiveness or behavioral sensitization remains unknown.

Sex differences in cocaine responses are also well documented (Fernandez-Espejo and Rodriguez-Espinosa, 2011; Zachry et al., 2021; Dos Anjos Rosário et al., 2022). Female rodents generally exhibit greater acute cocaine-induced locomotor activity and more rapid development of behavioral sensitization than males, effects that are strongly influenced by estradiol-mediated facilitation of mesolimbic dopamine signaling (Chin et al., 2001; Becker and Hu, 2008; Martínez-Caballero et al., 2025). Females also acquire cocaine self-administration more rapidly and exhibit greater motivation, escalation, and reinstatement of cocaine seeking compared with males (Lynch and Carroll, 1999; Carroll and Anker, 2010). In contrast, males generally display slower acquisition and lower overall responding across several paradigms (Hu et al., 2004; Jackson et al., 2006). Furthermore, GPCR signaling has been increasingly recognized as exhibiting sex-dependent regulation in the brain, highlighting biological sex as a critical determinant of GPCR function (Aljoudi et al., 2024). Whether genotype-dependent effects of GPR4 interact with sex and sensitization across temporally resolved cocaine responses has not been systematically examined.

The present study investigated the contribution of GPR4 to acute, repeated, and sensitized cocaine-induced locomotor responses in 3-month-old mice using high-resolution temporal analysis. We hypothesized that deletion of GPR4 would alter cocaine-induced locomotor behavior in a temporally dynamic manner and that these effects would interact with biological sex and prior cocaine exposure. By examining behavioral responses across acute and sensitized states, this study sought to determine whether proton-sensitive GPR4 signaling contributes to cocaine-induced neurobehavioral plasticity.

## 2 Materials and methods

### 2.1 Animals

Wild-type (WT) and GPR4 knockout (GPR4KO) mice were bred and maintained at the University of Missouri–Kansas City animal facility under controlled temperature and humidity conditions on a 12-h light/dark cycle with ad libitum access to food and water. Mice were group housed (3–5 animals per cage) under identical environmental conditions. Behavioral testing was conducted during the light phase.

A total of 166 mice (3 months of age) were used in this study. Among GPR4KO mice, 48 received cocaine (28 males and 20 females) and 41 received saline (26 males and 15 females). Among WT mice, 41 received cocaine (16 males and 25 females) and 36 received saline (13 males and 23 females) (Supplementary Table S1).

Mice were randomly assigned to experimental groups. Experimenters were blinded to genotype and treatment conditions during behavioral testing, data processing, and statistical analyses. To minimize potential litter and cage effects, animals from different litters were distributed across experimental groups whenever possible, and group allocation was balanced across litters when feasible.

Exclusion criteria were defined *a priori* and were limited to technical failure of the behavioral recording system or incomplete data acquisition. No animals were excluded based on behavioral outcomes.

All experimental procedures were approved by the University of Missouri–Kansas City Institutional Animal Care and Use Committee (IACUC) and were conducted in accordance with the National Institutes of Health Guide for the Care and Use of Laboratory Animals. GPR4KO mice were generously provided by Dr. Xiangming Zha (Tulane University).

### 2.2 Experimental design

#### 2.2.1 Behavioral testing protocol

The locomotor testing protocol was adapted from our previous study (Jiang et al., 2013). Each testing session lasted 150 min and consisted of a 60-min habituation period followed by intraperitoneal (i.p.) injection and 90 min of post-injection locomotor monitoring.

For each session, mice were placed individually into locomotor activity chambers and allowed to habituate for 60 min prior to drug administration. Following habituation, mice received either saline or cocaine, and locomotor activity was continuously recorded for an additional 90 min.

To assess acute and repeated cocaine responses, mice underwent one testing session per day for five consecutive days (Days 1–5). During this phase, mice received either saline or cocaine (20 mg/kg, i.p.) according to treatment group assignment.

Following completion of the repeated exposure phase, mice underwent a 14-day drug-free abstinence period to permit assessment of longer-term neuroadaptive changes. On the challenge day, all mice, including those previously treated with saline, received a cocaine challenge injection (10 mg/kg, i.p.) and were tested using the same 150-min locomotor protocol. The lower cocaine challenge dose was selected to assess the expression of locomotor sensitization following prior cocaine exposure.

#### 2.2.2 Locomotor activity and outcome measures

Locomotor activity was measured using the VersaMax Animal Activity Monitoring System (AccuScan Instruments, Columbus, OH, USA). Total distance traveled (cm) was recorded in consecutive 5-min intervals throughout each 150-min testing session.

The primary behavioral outcome during repeated cocaine exposure was post-injection locomotor activity during the 90-min recording period (5–90 min post-injection). Locomotor responses were analyzed both as time-resolved activity across individual 5-min intervals and as cumulative post-injection distance traveled. Time-resolved analyses were prespecified to characterize the temporal evolution of genotype-and sex-dependent cocaine responses, whereas cumulative post-injection activity provided an integrated measure of overall behavioral activation.

Behavioral sensitization was defined *a priori* as an enhanced locomotor response to the cocaine challenge in mice previously exposed to repeated cocaine relative to mice previously exposed to saline. Sensitization was quantified using cumulative post-injection locomotor activity and further characterized by analyses of the temporal pattern of locomotor responses during the challenge session. Estimated marginal means were used to quantify genotype-and sex-specific contrasts during sensitization testing.

#### 2.2.3 Statistical analysis

Statistical analyses were performed in R (version 4.4.1). Continuous locomotor activity was analyzed using linear mixed-effects models implemented with the lme4 package.

For repeated cocaine exposure (Days 1–5), locomotor activity was analyzed using linear mixed-effects models that included genotype and sex as between-subject factors and experimental day and post-injection time (5-min bins) as repeated within-subject factors. Mouse identity was included as a random intercept to account for repeated measurements obtained from the same animal.

For the cocaine challenge experiment, locomotor activity was analyzed using similar linear mixed-effects models that included genotype, sex, prior treatment history (previous cocaine versus previous saline exposure), and post-injection time as fixed effects, with mouse identity included as a random intercept.

Cumulative post-injection locomotor activity (5–90 min post-injection) was analyzed separately. During repeated cocaine exposure, cumulative activity was analyzed using linear mixed-effects models with genotype, sex, and experimental day as fixed effects and mouse identity as a random intercept. For the cocaine challenge experiment, cumulative activity was analyzed using factorial linear models that included genotype, sex, prior treatment history, and their interactions.

Because group sizes were unequal, primary statistical inference was based on Type III analyses of variance using sum-to-zero contrast coding. Model assumptions were evaluated by visual inspection of residual-versus-fitted plots, normal quantile-quantile plots, and residual distributions. Estimated marginal means and corresponding 95% confidence intervals were calculated using the emmeans package to facilitate interpretation of significant main effects and interactions. Pairwise comparisons were restricted to biologically relevant planned contrasts and were adjusted for multiple testing using the Benjamini-Hochberg false discovery rate procedure. Effect sizes are reported as Hedges’ g with corresponding 95% confidence intervals. Statistical significance was defined as a two-sided adjusted *p* < 0.05.

Sensitivity analyses using Type II analyses of variance, heteroscedasticity-consistent (HC3) standard errors, and log1p-transformed locomotor activity yielded conclusions consistent with the primary analyses, confirming the robustness of the reported findings.

## 3 Results

### 3.1 Baseline locomotor activity during the acclimation period

Baseline locomotor activity was recorded during the 60-min pre-injection acclimation period before saline or cocaine administration. Across Days 1–5, analysis of mean baseline locomotor activity revealed significant main effects of genotype (*F* = 9.89, *p* = 0.0023) and sex (*F* = 12.19, *p* = 0.00076), with no significant genotype × sex interaction (*p* > 0.05). GPR4KO mice exhibited greater baseline locomotor activity than WT mice, and females displayed greater baseline activity than males (Supplementary Figure S1). *Post hoc* comparisons confirmed higher baseline activity in GPR4KO mice compared with WT mice in both females (*p* = 0.030) and males (*p* = 0.026). In addition, females exhibited higher baseline locomotor activity than males in both WT (*p* = 0.032) and GPR4KO (*p* = 0.0066) groups. These results indicate that genotype and sex independently influence spontaneous locomotor activity during the acclimation period.

Following the 14-day abstinence period, baseline locomotor activity during the 60-min pre-injection acclimation period on the final test day was influenced by genotype and sex. Three-way ANOVA revealed significant main effects of genotype (*p* = 0.040) and sex (*p* = 0.0023), as well as a significant genotype × sex × prior treatment interaction (*p* = 0.0049). In contrast, the main effect of prior treatment history was not significant, indicating that repeated cocaine exposure did not produce an overall change in pre-challenge baseline locomotor activity. *Post hoc* analyses showed that GPR4KO mice exhibited greater baseline activity than WT mice in males previously treated with saline (*p* = 0.033) and females previously treated with cocaine (*p* = 0.014), but no genotype differences were detected in females previously treated with saline or males previously treated with cocaine (Supplementary Figure S2). Collectively, these findings indicate that pre-challenge baseline locomotor activity is determined by genotype and sex, with the effect of genotype differing according to prior cocaine exposure history.

### 3.2 Acute cocaine-induced locomotor activity during repeated exposure

**Figure 1:**
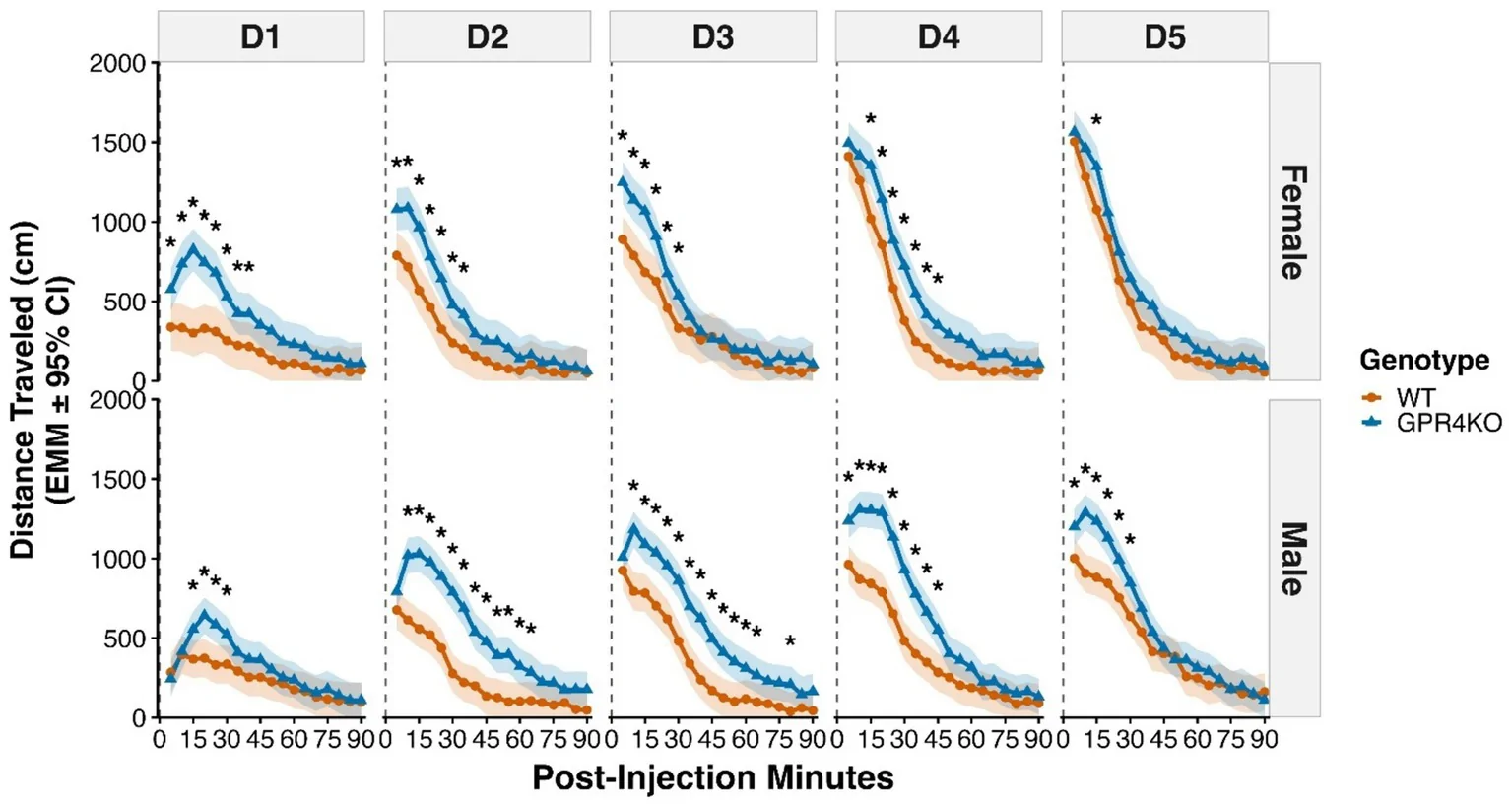
Acute cocaine-induced total distance traveled during repeated cocaine exposure. Repeated cocaine administration (20 mg/kg, i.p.) produced progressive locomotor sensitization across Days 1–5. Estimated marginal means (EMMs ± 95% confidence intervals [CIs]) from the linear mixed-effects model are shown for total distance traveled (cm), measured in 5-min bins during the 5–90 min post-injection period. Panels are stratified by sex and day of cocaine exposure. Asterisks indicate adjusted *p* < 0.05 for comparisons between WT and GPR4KO mice at individual time bins. Cocaine was administered at 0 min. Lines represent estimated marginal means, and shaded regions represent 95% confidence intervals.

Repeated cocaine administration (20 mg/kg) produced progressive increases in locomotor activity across Days 1–5 in both WT and GPR4KO mice, reflecting progressive locomotor adaptation during repeated cocaine exposure (Figure 1). Linear mixed-effects analysis of post-injection locomotor activity revealed significant main effects of genotype (*F* = 32.09, *p* < 0.0001), day (*F* = 186.41, *p* < 0.0001), and post-injection time (*F* = 585.68, *p* < 0.0001). GPR4KO mice exhibited greater locomotor activity than WT mice throughout the repeated cocaine exposure period. Significant genotype × day (*F* = 10.67, *p* < 0.0001) and genotype × time (*F* = 17.14, *p* < 0.0001) interactions indicated that genotype-dependent differences varied across repeated exposure days and within individual testing sessions. Although the overall genotype × sex interaction was not significant (*F* = 0.35, *p* = 0.554), a significant genotype × sex × day interaction (*F* = 12.70, *p* < 0.0001) demonstrated that the progression of genotype-dependent locomotor responses differed between males and females (Figure 1).

Time-resolved *post hoc* comparisons further delineated the temporal profile of genotype effects across the post-injection period. In females, GPR4KO mice exhibited significantly greater locomotor activity than WT mice during 5–40 min on Day 1, 5–35 min on Day 2, 5–30 min on Day 3, 15–45 min on Day 4, and at 15 min on Day 5. In males, significant genotype differences were observed during 15–30 min on Day 1, 10–65 min on Day 2, 10–65 min and again at 80 min on Day 3, 5–45 min on Day 4, and 5–30 min on Day 5. In every significant interval, GPR4KO mice exhibited greater locomotor activity than WT mice. Overall, genotype effects were most prominent during the early-to-intermediate post-injection phase and were more prolonged in males than in females, particularly during Days 2–4.

**Figure 2:**
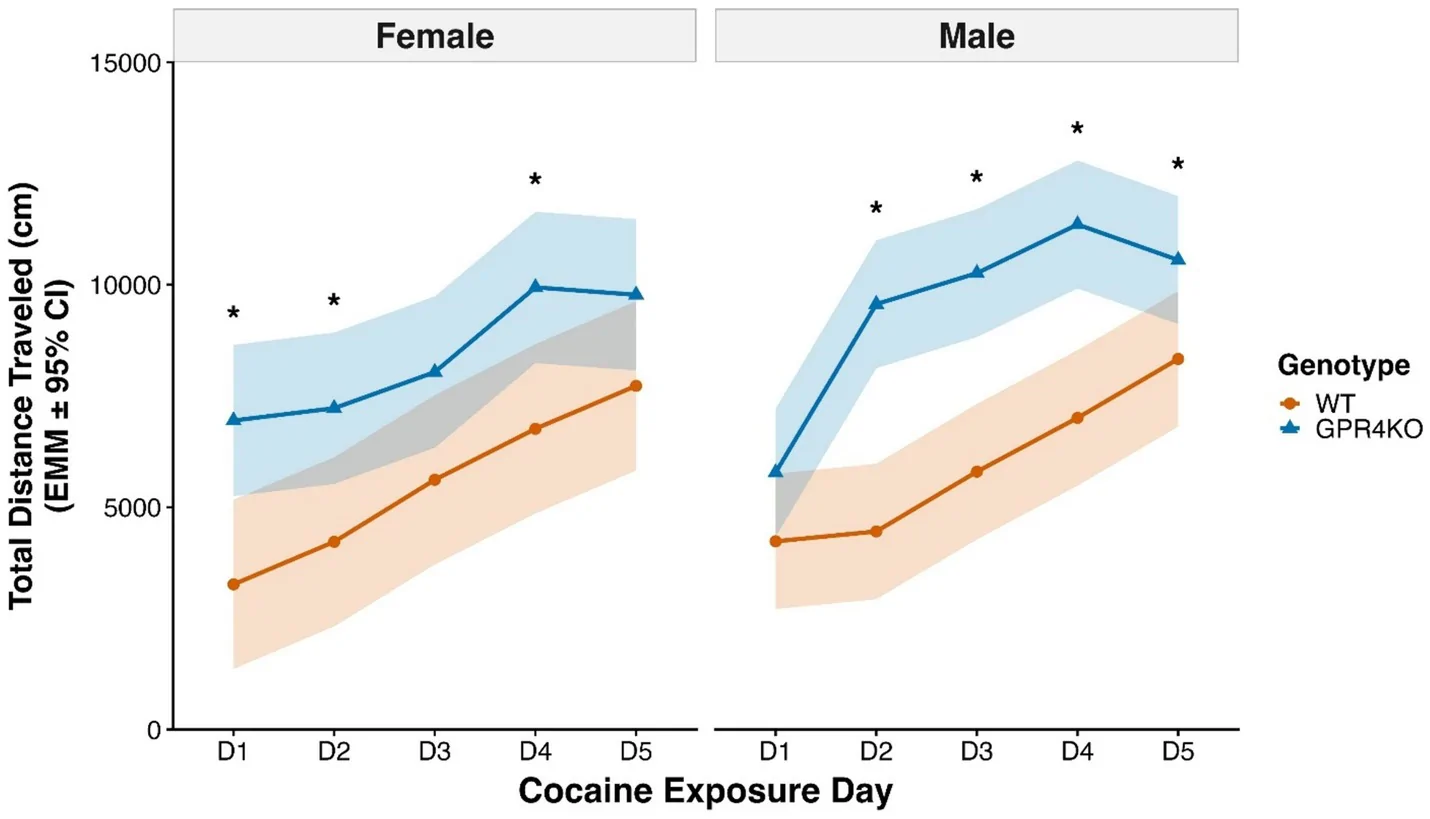
Acute cumulative total distance traveled during repeated cocaine exposure. Total distance traveled (cm) during the 5–90 min post-injection period following repeated cocaine administration (20 mg/kg, i.p.) across Days 1–5. Estimated marginal means (EMMs ± 95% confidence intervals [CIs]) from the linear mixed-effects model are shown and are stratified by sex and day of cocaine exposure. Asterisks indicate adjusted *p* < 0.05 for comparisons between WT and GPR4KO mice within each cocaine exposure day. Points represent estimated marginal means, and error bars represent 95% CIs.

Analysis of cumulative post-injection locomotor activity (5–90 min post-injection) confirmed significant main effects of genotype (*F* = 32.09, *p* < 0.0001) and day (*F* = 25.73, *p* < 0.0001), with greater overall locomotor activity in GPR4KO mice and progressive increases across repeated cocaine exposure (Figure 2). In contrast to the time-resolved analysis, cumulative activity did not reveal significant genotype × day (*F* = 1.47, *p* = 0.210) or genotype × sex × day (*F* = 1.75, *p* = 0.138) interactions, indicating that sex-dependent temporal differences in locomotor responses were primarily evident in the within-session pattern rather than cumulative activity.

*Post hoc* comparisons revealed sex-specific patterns of genotype effects across repeated cocaine exposure. In females, GPR4KO mice exhibited greater cumulative locomotor activity than WT mice on Day 1 (*Δ* = −3682.6 cm, 95% confidence interval [CI] = −6232.0 to −1133.1; adjusted *p* = 0.0048; Hedges’ *g* = −1.20), Day 2 (Δ = −3002.4 cm, 95% CI = −5530.6 to −474.1; adjusted *p* = 0.021; *g* = −0.98), and Day 4 (*Δ* = −3179.1 cm, 95% CI = −5707.3 to −650.8; adjusted *p* = 0.015; *g* = −1.04). Genotype differences were not significant on Day 3 (adjusted *p* = 0.063) or Day 5 (adjusted *p* = 0.115).

In males, genotype differences were smaller on Day 1 (*Δ* = −1545.4 cm, 95% CI = −3633.8 to 543.0; adjusted *p* = 0.147; *g* = −0.45) but increased during subsequent cocaine exposures. Significant genotype differences were observed on Day 2 (Δ = −5108.1 cm, 95% CI = −7117.6 to −3098.7; adjusted *p* = 2.4 × 10^−6^; *g* = −1.67), Day 3 (Δ = −4462.1 cm, 95% CI = −6471.6 to −2452.7; adjusted *p* = 3.5 × 10^−5^; *g* = −1.46), Day 4 (Δ = −4347.5 cm, 95% CI = −6357.0 to −2338.1; adjusted *p* = 5.5 × 10^−5^; *g* = −1.42), and Day 5 (Δ = −2222.1 cm, 95% CI = −4231.5 to −212.6; adjusted *p* = 0.037; *g* = −0.73).

Together, these findings demonstrate that GPR4 deficiency enhances cocaine-induced locomotor responses during repeated exposure and that genotype-dependent effects exhibit distinct temporal patterns in male and female mice.

### 3.3 Expression of cocaine-induced locomotor sensitization following abstinence

**Figure 3:**
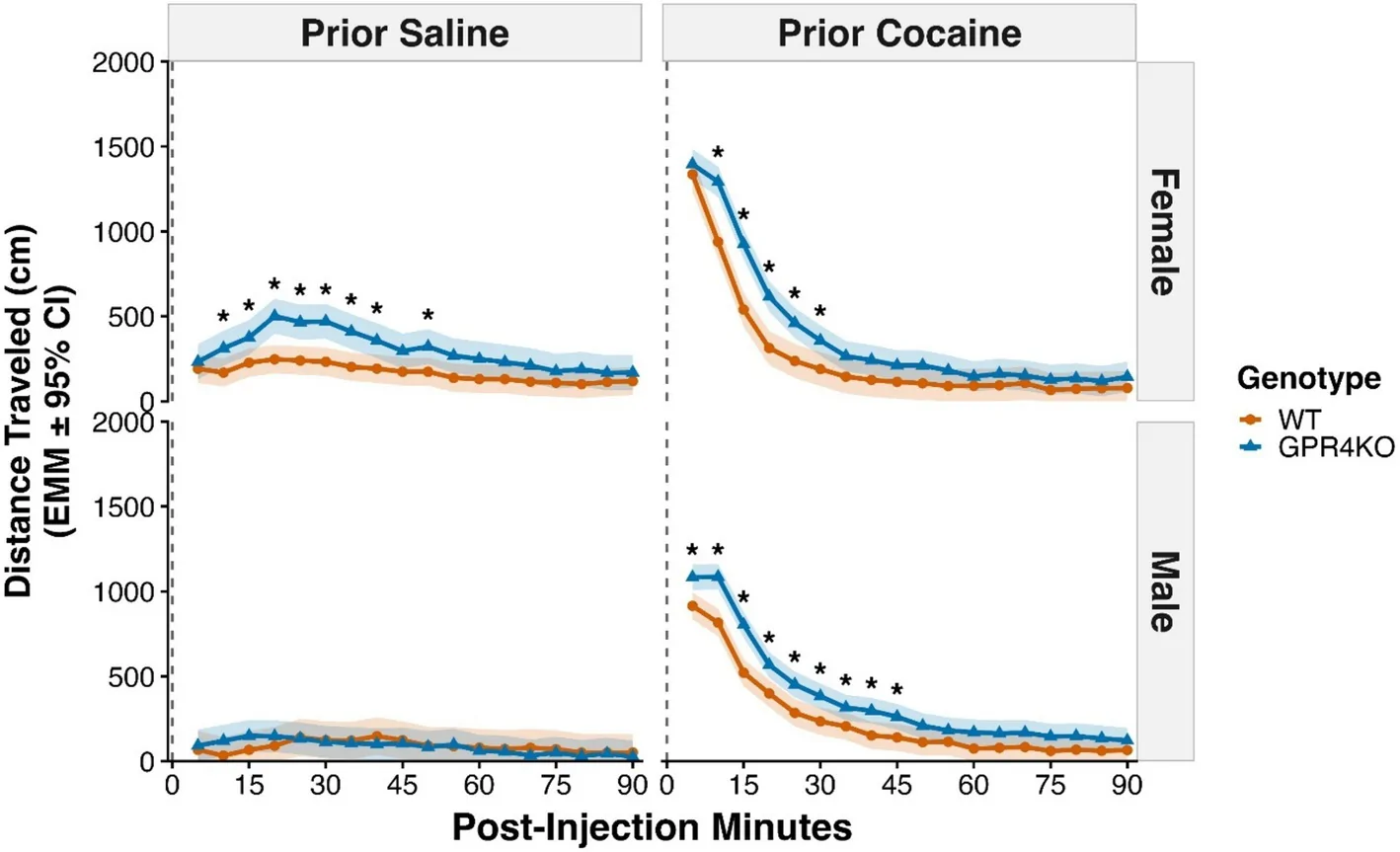
Expression of cocaine-induced locomotor sensitization after a 14-day abstinence period. Following a 14-day abstinence period, mice previously exposed to cocaine or saline received a cocaine challenge (10 mg/kg, i.p.) to assess the expression of locomotor sensitization. Estimated marginal means (EMMs ± 95% confidence intervals [CIs]) from the linear mixed-effects model are shown for total distance traveled (cm), measured in 5-min bins during the 5–90 min post-injection period. Panels are stratified by prior treatment group and sex. Asterisks indicate adjusted *p* < 0.05 for comparisons between WT and GPR4KO mice at individual time bins. Cocaine challenge was administered at 0 min. Lines represent estimated marginal means, and shaded regions represent 95% CIs.

Following the 14-day abstinence period, all mice received a cocaine challenge injection (10 mg/kg) to assess the expression of locomotor sensitization (Figure 3). Linear mixed-effects analysis of post-injection locomotor activity revealed significant main effects of genotype (*F* = 22.55, *p* < 0.0001), sex (*F* = 16.52, *p* < 0.0001), prior treatment history (previous cocaine versus previous saline exposure; *F* = 58.06, *p* < 0.0001), and post-injection time (*F* = 518.91, *p* < 0.0001). These findings indicate that locomotor responses during the cocaine challenge were influenced by genotype, sex, prior cocaine exposure, and the temporal progression of the cocaine response. A significant sex × prior treatment interaction (*F* = 9.06, *p* = 0.003) demonstrated that the magnitude of sensitization differed between males and females. In contrast, genotype × prior treatment (*F* = 2.37, *p* = 0.125), genotype × sex (*F* = 0.31, *p* = 0.579), and genotype × sex × prior treatment (*F* = 0.64, *p* = 0.426) interactions were not significant (Figure 3).

Time-resolved *post hoc* comparisons revealed distinct temporal patterns of genotype effects across sex and sensitization history. In females, GPR4KO mice exhibited greater locomotor activity than WT mice following both prior cocaine exposure (10–30 min) and prior saline exposure (10–40 min and 50 min). In contrast, among males, significant genotype differences were observed only in the cocaine-exposed group, where GPR4KO mice displayed elevated locomotor activity from 5–45 min after the challenge; no significant differences were detected in saline-treated males. Together, these findings suggest that prior cocaine exposure selectively prolonged genotype-dependent differences in males, whereas genotype effects in females were evident regardless of sensitization history.

**Figure 4:**
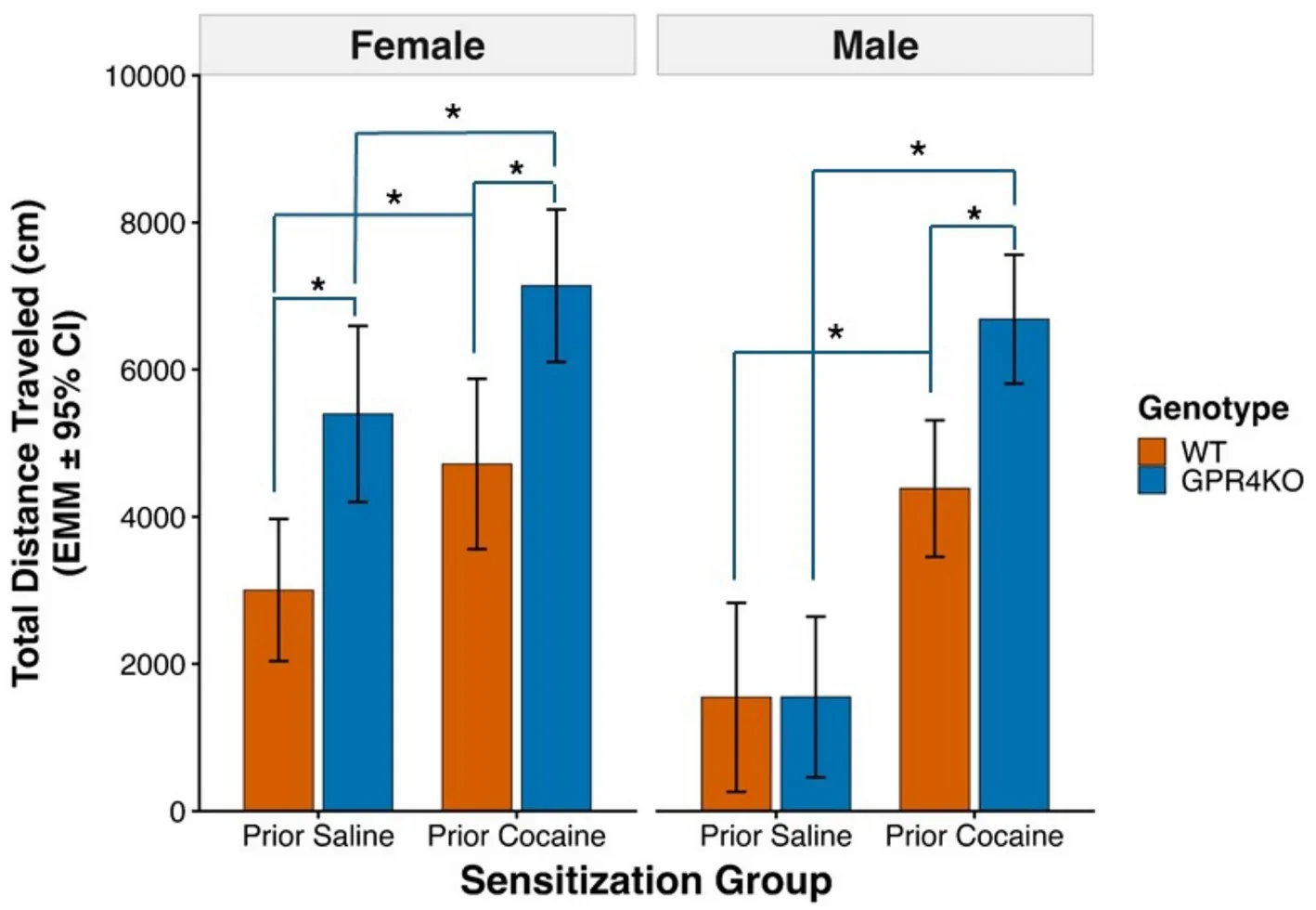
Cumulative total distance traveled during the cocaine challenge. Total distance traveled (cm) during the 5–90 min post-injection period following a cocaine challenge (10 mg/kg, i.p.) after a 14-day abstinence period. Bars represent estimated marginal means (EMMs ± 95% confidence intervals [CIs]) from the linear mixed-effects model and are stratified by prior treatment group and sex. Asterisks indicate adjusted *p* < 0.05 for pairwise comparisons between genotypes and between sexes (within WT and GPR4KO mice) for each prior treatment group.

Analysis of cumulative post-injection locomotor activity confirmed robust expression of sensitization across genotype and sex groups (Figure 4). Compared with mice that received prior saline exposure, mice with prior cocaine exposure exhibited significantly greater locomotor activity following the cocaine challenge in WT males (*Δ* = 2,837.0 cm, 95% CI = 1,279.4–4,394.6 cm; adjusted *p* = 5.4 × 10^−4^; Hedges’ *g* = 1.13), GPR4KO males (Δ = 5,135.2 cm, 95% CI = 3,677.2–6,593.2 cm; adjusted *p* = 2.1 × 10^−11^; *g* = 2.02), WT females (*Δ* = 1,714.3 cm, 95% CI = 215.0–3,213.7 cm; adjusted *p* = 0.026; *g* = 0.76), and GPR4KO females (Δ = 1,742.9 cm, 95% CI = 170.4–3,315.3 cm; adjusted *p* = 0.031; *g* = 0.75). Thus, repeated cocaine exposure produced significant sensitized locomotor responses in all genotype and sex groups.

Planned pairwise comparisons further examined genotype differences within each sex and sensitization condition. Among females, WT and GPR4KO mice exhibited comparable sensitization magnitudes (Δ = 28.6 cm, 95% CI = −2,048.2 to 2,105.4 cm; adjusted *p* = 0.979; Hedges’ *g* = 0.01). Among males, GPR4KO mice showed a greater sensitized response than WT mice (Δ = 2,298.2 cm, 95% CI = 188.2–4,408.3 cm; adjusted *p* = 0.033; *g* = 0.98). Consistent with these findings, genotype differences during the cocaine challenge were more pronounced in males with prior cocaine exposure, whereas female genotype differences remained similar regardless of prior treatment history (Figures 3, 4).

Together, these findings demonstrate robust expression of locomotor sensitization following repeated cocaine exposure and indicate that sex influences the magnitude of sensitized responses. Although the overall genotype × sensitization interaction was not significant, exploratory genotype comparisons suggest that GPR4-associated differences may be more apparent in males following repeated cocaine exposure.

## 4 Discussion

CUD remains a major public health challenge, and effective pharmacological treatments remain limited (Chan et al., 2019; Kampman, 2019; Bentzley et al., 2021; Brandt et al., 2021). Cocaine-induced behavioral adaptations arise from complex interactions among genetic factors, neural circuits, and environmental influences that produce persistent neuroplastic changes within addiction-related pathways (Breiter et al., 1997; Nestler, 2001; Nestler, 2025; Carbone et al., 2026). Identifying molecular regulators that influence cocaine-induced behavioral responses may provide new insights into mechanisms underlying addiction vulnerability and resilience.

In this study, we identify the proton-sensing G protein-coupled receptor GPR4 as a previously unrecognized regulator of cocaine-induced behavioral adaptations. Genetic deletion of GPR4 increased basal locomotor activity, enhanced acute cocaine-induced locomotor activation, and altered the temporal pattern of behavioral responses during repeated cocaine exposure and subsequent sensitization testing. Importantly, these effects were influenced by biological sex and cocaine exposure history, demonstrating that GPR4 contributes to the dynamic regulation of cocaine-related behavioral plasticity.

### 4.1 GPR4 regulates basal locomotor activity

A major finding of this study is that GPR4 contributes to the regulation of spontaneous locomotor activity under drug-free conditions. During the repeated exposure phase, GPR4KO mice exhibited higher baseline locomotor activity than WT mice during the pre-injection habituation period. Female mice also displayed greater baseline activity than males, consistent with previous reports describing sex differences in exploratory behavior and locomotor activity. The absence of a genotype × sex interaction suggests that the effect of GPR4 deletion on basal locomotion is broadly similar between males and females.

Following the 14-day abstinence period, genotype and sex continued to influence pre-challenge baseline activity, whereas prior cocaine exposure alone did not produce persistent increases in spontaneous locomotion. These findings indicate that repeated cocaine exposure does not uniformly alter basal activity after withdrawal and suggest that GPR4 contributes to regulation of baseline behavioral state independent of cocaine administration.

The elevated baseline activity observed in GPR4KO mice is an important consideration when interpreting cocaine-induced locomotor responses. Although differences in basal activity may contribute to differences in absolute locomotor distance, genotype-dependent effects remained evident following cocaine administration and exhibited distinct temporal patterns across repeated exposure. Thus, GPR4 appears to regulate both general locomotor activity and cocaine-induced behavioral activation.

### 4.2 GPR4 modulates acute cocaine-induced locomotor activation in a time-and sex-dependent manner

The primary finding of this study is that GPR4 deletion enhanced cocaine-induced locomotor activation. Across repeated cocaine administration, GPR4KO mice consistently exhibited greater locomotor responses than WT mice, suggesting that endogenous GPR4 signaling normally constrains cocaine-induced behavioral activation.

Importantly, GPR4 deletion did not simply produce a uniform increase in cocaine responsiveness. Instead, genotype effects varied across repeated exposure days and within individual behavioral sessions. While cumulative locomotor activity demonstrated overall higher responses in GPR4KO mice, time-resolved analyses revealed that genotype differences were dynamically organized across the cocaine response profile. These findings highlight the importance of temporal analysis when evaluating complex behavioral adaptations and suggest that GPR4 may influence the initiation, magnitude, and duration of cocaine-induced behavioral activation.

Sex-dependent patterns further demonstrate that GPR4 regulation of cocaine responses is not identical between males and females. Female GPR4KO mice showed greater genotype differences during early cocaine exposure, whereas genotype differences became less pronounced as WT females increased their responses with repeated administration. In contrast, males showed greater genotype divergence during intermediate and later exposure sessions. These distinct trajectories suggest that biological sex modifies how GPR4 signaling influences cocaine-induced behavioral adaptations.

The mechanisms underlying these sex-dependent patterns remain unknown. Differences in dopamine signaling, hormone regulation, synaptic plasticity, and stress-related pathways may contribute to the divergent behavioral trajectories observed between males and females. Nevertheless, these findings demonstrate that GPR4 effects cannot be fully understood without considering sex as an important biological variable.

### 4.3 GPR4 influences cocaine-induced locomotor sensitization after abstinence

Following a 14-day abstinence period, both WT and GPR4KO mice exhibited robust expression of locomotor sensitization after cocaine challenge, demonstrating that repeated cocaine exposure produced persistent behavioral adaptations that were maintained after withdrawal. Prior cocaine exposure increased locomotor responses in all genotype and sex groups, indicating that the development and expression of sensitization can occur in the absence of GPR4.

However, GPR4 deletion increased overall cocaine-induced locomotor activity during the challenge session, indicating that GPR4 continues to regulate cocaine responsiveness after prolonged abstinence. Although the genotype × sensitization interaction was not statistically significant, planned comparisons suggested that genotype-related differences in sensitized responses may be more apparent in males than females. These findings should be interpreted cautiously but raise the possibility that GPR4 contributes to persistent cocaine-induced adaptations in a sex-dependent manner.

The significant sex × sensitization interaction further emphasizes the importance of biological sex in determining the magnitude of cocaine-induced neurobehavioral plasticity. Consistent with previous studies, females in the present study exhibited greater cocaine-induced locomotor activity during sensitization testing than males. Female rodents frequently display enhanced psychostimulant responsiveness and more rapid development of behavioral sensitization, effects that have been attributed, in part, to estradiol-mediated facilitation of mesolimbic dopamine signaling and associated synaptic adaptations (Hu and Becker, 2003; Becker and Hu, 2008; Becker and Chartoff, 2019). Our findings extend these observations by demonstrating that sex differences in cocaine responsiveness are also influenced by GPR4 genotype and exhibit temporally dynamic features during repeated cocaine exposure and abstinence. Although both sexes developed robust sensitized responses, genotype-related differences during sensitization were more apparent in males, suggesting that biological sex may differentially modulate proton-sensitive signaling mechanisms that contribute to cocaine-induced behavioral adaptations. Together, these findings reinforce the importance of incorporating sex as a biological variable in addiction research (Fattore et al., 2020).

### 4.4 Potential mechanisms linking GPR4 to cocaine-induced behavioral plasticity

The present study identifies a behavioral role for GPR4 but does not directly determine the cellular mechanisms responsible for these effects. GPR4 is activated by extracellular acidification and regulates intracellular signaling pathways, including Gs/cAMP-dependent signaling and cellular stress responses (Ludwig et al., 2003). Because cocaine increases neuronal activity, dopamine release, and metabolic demand within mesocorticolimbic circuits, it may alter the extracellular microenvironment, including local pH dynamics. Proton-sensitive signaling through GPR4 may therefore represent a mechanism linking cocaine-induced neural activity with adaptive cellular responses.

Previous studies have established important roles for dopamine transmission, glutamatergic plasticity, and intracellular signaling pathways in cocaine-induced behavioral adaptations (Wolf, 1998; Kalivas, 2009; Nestler, 2025). Although GPR4 signaling may interact with these pathways, the present study did not directly measure dopamine function, synaptic plasticity, neuronal activity, or extracellular pH changes. Therefore, the proposed relationship between GPR4 and cocaine-induced neuroplasticity remains mechanistic speculation requiring further investigation.

Future studies combining behavioral analysis with neurochemical, electrophysiological, and cell type-specific approaches will be important for determining how GPR4 influences mesocorticolimbic circuit function.

### 4.5 Limitations and future directions

Several limitations should be considered. First, locomotor sensitization provides an indirect measure of cocaine-induced behavioral plasticity and does not directly assess reward, motivation, or compulsive drug-seeking behaviors. Future studies using cocaine self-administration, conditioned place preference, extinction, and reinstatement paradigms will determine whether GPR4 contributes to broader addiction-related behaviors.

Second, the present study used global GPR4 deletion and therefore cannot identify the specific neural populations or cellular mechanisms responsible for the observed phenotype. Conditional genetic approaches and circuit-specific manipulation will be necessary to determine the contribution of neuronal versus non-neuronal GPR4 signaling.

Third, estrous cycle stage was not monitored in female mice. Because ovarian hormones influence cocaine responsiveness and mesolimbic dopamine signaling (Hu and Becker, 2003; Souza et al., 2014; Knouse and Briand, 2021), future studies incorporating hormonal staging will be important for defining the contribution of sex hormones to GPR4-dependent behavioral regulation.

### 4.6 Conclusion

In summary, this study identifies GPR4 as a novel regulator of cocaine-induced behavioral responses. GPR4 deletion increases basal locomotor activity, enhances acute cocaine-induced locomotor activation, and modifies the temporal organization of cocaine-induced behavioral adaptations following repeated exposure and abstinence. These effects are influenced by sex and cocaine exposure history, highlighting the complex and dynamic regulation of cocaine responses by proton-sensitive signaling mechanisms. As a proton-sensing GPCR, GPR4 may represent an important molecular link between extracellular pH regulation and cocaine-induced neurobehavioral plasticity. Further investigation of GPR4 function within addiction-related neural circuits may reveal new mechanisms contributing to cocaine-induced behavioral adaptations.

## Data Availability

The original contributions presented in the study are included in the article/Supplementary material, further inquiries can be directed to the corresponding author.

## Supplementary material

SUPPLEMENTARY FIGURE S1 Baseline pre-injection total distance traveled during repeated cocaine exposure. Baseline pre-injection total distance traveled during repeated cocaine exposure in 3-month-old mice. Total distance traveled (cm) during the 0–60 min pre-injection acclimation period was averaged across Days 1–5 for each mouse and analyzed by genotype and sex. Bars represent estimated marginal means (EMMs ± 95% confidence intervals [CIs]) from the statistical model. Asterisks indicate adjusted *p* < 0.05 for pairwise comparisons between genotypes and between sexes (within WT and GPR4KO mice).

SUPPLEMENTARY FIGURE S2 Baseline pre-injection total distance traveled before cocaine challenge after abstinence. Baseline pre-injection total distance traveled before the final cocaine challenge in 3-month-old mice. Total distance traveled (cm) during the 0–60 min pre-injection acclimation period before the final cocaine challenge was analyzed by genotype, sex, and prior treatment history. Prior saline and prior cocaine groups indicate treatment history during the initial cocaine exposure phase before the 14-day abstinence period and final cocaine challenge. Bars represent estimated marginal means (EMMs ± 95% confidence intervals [CIs]) from the statistical model. Asterisks indicate adjus ted *p* < 0.05 for pairwise comparisons between WT and GPR4KO mice within each sex and prior treatment group.
